# Supplementary material for: Inflammatory factors and risk of lung adenocarcinoma: a Mendelian randomization study mediated by blood metabolites
Source: Front Endocrinol (Lausanne). 2024 Aug 27;15:1446863. doi: 10.3389/fendo.2024.1446863 (PMC11384989; doi:10.3389/fendo.2024.1446863)
Supplement: Supplementary file 1 [file DataSheet1.zip › supplementary files/S2/GCST90200994/sensitivity-analysis.pdf]

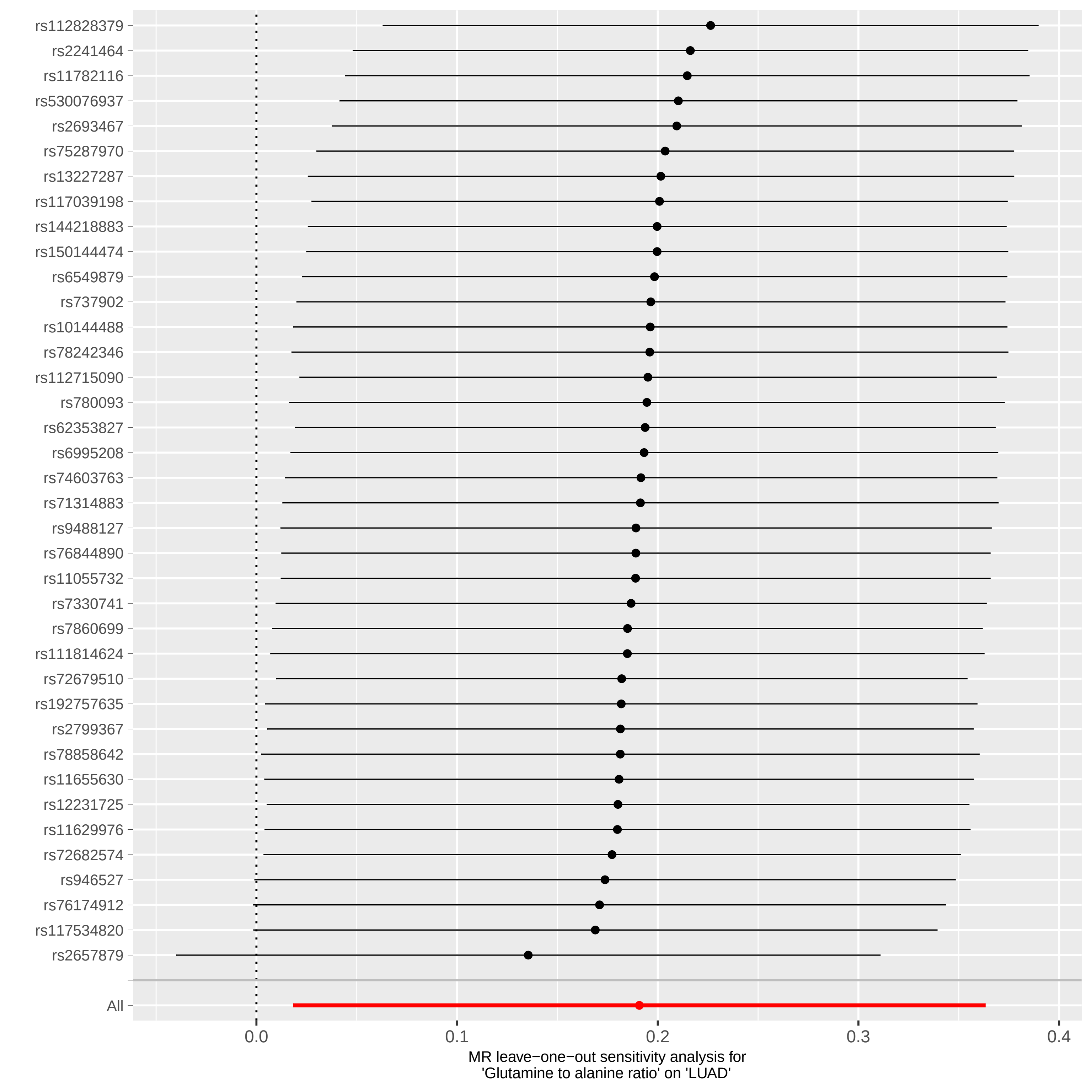
rs112828379
rs2241464
rs11782116
rs530076937
rs2693467
rs75287970
rs13227287
rs117039198
rs144218883
rs150144474
rs6549879
rs737902
rs10144488
rs78242346
rs112715090
rs780093
rs62353827
rs6995208
rs74603763
rs71314883
rs9488127
rs76844890
rs11055732
rs7330741
rs7860699
rs111814624
rs72679510
rs192757635
rs2799367
rs78858642
rs11655630
rs12231725
rs11629976
rs72682574
rs946527
rs76174912
rs117534820
rs2657879
All
0.0
0.1
0.2
0.3
0.4
MR leave−one−out sensitivity analysis for
'Glutamine to alanine ratio' on 'LUAD'
